# Visuospatial cognition in people with Parkinson’s disease: a pilot study assessing a block span task using fMRI

**DOI:** 10.3389/fnhum.2026.1762612

**Published:** 2026-03-25

**Authors:** Emily Z. Zhang, Gaelle E. Doucet, Daniel Huddleston, Anastasia A. Bohsali, J. Lucas Mckay, Venkatagiri Krishnamurthy, Madeleine E. Hackney

**Affiliations:** 1 Duke University School of Medicine, Durham, NC, United States; 2 Emory College of Arts and Sciences, Atlanta, GA, United States; 3 Institute for Human Neuroscience, Boys Town National Research Hospital, Boys Town, NE, United States; 4 Center for Pediatric Brain Health, Boys Town National Research Hospital, Boys Town, NE, United States; 5 Department of Pharmacology and Neuroscience, School of Medicine, Creighton University, Omaha, NE, United States; 6 Department of Neurology, Emory University School of Medicine, Atlanta, GA, United States; 7 Tri-institutional Center for Translational Research in Neuroimaging and Data Science (TReNDS), Georgia State University, Atlanta, GA, United States; 8 Department of Biomedical Informatics, Emory University School of Medicine, Atlanta, GA, United States; 9 Department of Biomedical Engineering, Georgia Tech and Emory University, Atlanta, GA, United States; 10 United States Department of Veterans Affairs (VA) Health Care System, Rehabilitation R&D Center, Decatur, GA, United States; 11 Division of Geriatrics and Gerontology, Emory University School of Medicine, Atlanta, GA, United States; 12 Geriatric Research Education and Clinical Center, Birmingham/Atlanta VA, Brookhaven, GA, United States; 13 Department of Rehabilitation Medicine, Emory University School of Medicine, Atlanta, GA, United States

**Keywords:** Corsi task, encoding, fMRI, Parkinson’s disease, visuospatial, working memory

## Abstract

**Background:**

Impaired visuospatial working memory (WM) often negatively impacts the quality of life of patients with Parkinson’s Disease (PD). The Block Span Task (BST) is a novel Corsi-Block-like task adapted for use in functional magnetic resonance imaging (MRI) scanner to understand the neurophysiological mechanisms underlying visuospatial impairment in PD. This pilot study examines whether the BST is a practical tool to provide an in-scanner visuospatial WM task for older adults with mild–moderate PD.

**Methods:**

We recruited and assessed 21 older adults with mild–moderate PD (*F* = 9, *M* = 12, age: 70 ± 8 years, Hoehn and Yahr stage: 2.31 ± 0.54), with no overt dementia for participation in the task while lying in the scanner. They abstained from their anti-Parkinsonian medications for 12 h before the assessment. In scanner, participants viewed sequences of block locations on the screen before replicating the sequences on the corresponding buttons of a response pad. Following independent component analysis, components with positive, significant beta values were identified.

**Results:**

Administration of the BST task in an older PD population shows feasibility. Results from the encoding phase of the task were aligned with its intended design, implicating recruitment of neural networks with justifiable visuospatial WM involvement. The encoding phase of the task implicated recruitment of the superior temporal gyrus, superior medial frontal gyrus, precuneus, and posterior cingulate cortex, including auditory, cognitive, and default-mode networks.

**Conclusion:**

This pilot study provides initial evidence that impaired patients with PD can complete the BST fMRI task. Further, the BST appeared to engage significant neural regions that were consistent with an encoding condition in this population. Despite having a small sample size, these preliminary findings support that BST may be useful in future examination of visuo-spatial skills and the underlying brain mechanisms in patients with PD. Future works will be needed to replicate and refine these coarse and preliminary observations, and explore the degree of functional alterations in the recruited networks, when compared to healthy individuals.

## 1 Introduction

Often arising before motor symptoms in Parkinson’s disease (PD), nonmotor symptoms can be as distressing as motor symptoms. Cognitive impairment is a prominent non-motor symptom thought to significantly impair function and quality of life (QOL) in individuals with PD (Aarsland et al., 2021). Notably, cognitive deficits can further induce or exacerbate motor symptoms including falls as well as impaired driving and activities of daily life (França et al., 2023).

Visuospatial impairment is a common deficit in PD (Sezgin et al., 2019). Visuospatial working memory (WM) allows temporary storage and mental manipulation of visual information like object identity and location. This information is stored in the process of encoding and utilized through retrieval. Ability to mentally manipulate images is crucial to navigating physical environments because the visual appearance of objects continually changes as a person moves through a space and their perspective of the objects shifts. Thus, visuospatial WM is needed to hold a stable mental representation of an environment despite the dynamic nature of visual input. Thus, visuospatial deficits impair a person’s perception of their own spatial orientation and their ability to maintain balance (McAfoose and Baune, 2009). This impairment may contribute to the motor difficulties seen in PD, increasing PD patients’ risk of falling, bumping into objects, getting lost, or even having car accidents (França et al., 2023).

While the neuropathophysiology of PD has classically focused on the disrupted networks concerning the striatal and pallidal regions of the basal ganglia (BG) and neighboring structures, neuroimaging studies in PD have also implicated structural abnormalities as a result of the disease in regions beyond the BG including frontal, occipital, parietal, and whole brain atrophy (Sezgin et al., 2019). Several brain regions may specifically be involved in abnormalities of visuospatial function. Studies in people with PD have shown an association between performance on visuospatial tests and neurophysiological changes such as blunted glutamate response in the occipital cortex (Ophey et al., 2023) or atrophy in frontal and parietotemporal regions (Wylie et al., 2023). Functional MRI (fMRI) studies have associated visuospatial impairment in PD with altered activation in the prefrontal cortex, middle frontal gyrus, parietal lobule (Kawashima et al., 2021), cerebellum (Sako et al., 2021), BG, and limbic system (Caproni et al., 2014). These studies have potential in identifying key biomarkers of cognitive decline in PD.

Adapting established visuospatial assessment tasks to be compatible with simultaneous neuroimaging would allow studies to take advantage of the preexisting body of research on task performance. Diagnosis assessments for PD with cognitive impairment are not often initiated until after concerns regarding cognitive decline arise. Brain imaging including fMRI confirmation of the specific neuroanatomical involvement of visuospatial impairments could allow earlier identification and prevention of cognitive decline and present targets for future therapies (Wylie et al., 2023). A popular test of visuospatial WM in clinical and experimental contexts is the Corsi Block- Tapping Task which has been used in the assessment of several neurodegenerative conditions including Alzheimer’s Disease (Kessels et al., 2008). In PD, the Corsi task has demonstrated significant impairment of spatial short-term memory (Robbins et al., 1994) and reflected the progression of visuospatial deficits over time (Ramos et al., 2022). Previous fMRI studies of PD patients have adapted other visuospatial tasks for fMRI study of task-specific brain activation (Caproni et al., 2014; Kawashima et al., 2021; Sako et al., 2021). Adapting the Corsi blocks for fMRI in PD would facilitate further examination of the neural mechanisms of visuospatial cognition.

Several groups have previously adapted Corsi-like tasks for MRI studies of other populations. Nemmi et al. (2013) showed healthy participants, while in the MRI machine, a video clip of an experimenter demonstrating a pattern on a physical Corsi board and asked them to identify the correct pattern among three subsequent video clips showing different patterns. Toepper et al. (2010b) instead presented healthy participants in the MRI machine with a virtual replica of the Corsi board and asked them to replicate an illuminated pattern by making a series of forced-choice recognition responses between two different block positions. The groups demonstrated involvement of several regions during encoding, including the left inferior temporal gyrus, lingual and fusiform gyrus, and middle occipital gyrus in one study (Nemmi et al., 2013), or the right hippocampus and broad parietal, frontal, and occipital networks in another (Toepper et al., 2010a). Doucet et al. (2013) was among the first to use an adapted Corsi task in a patient population and to use a response pad for participants to replicate sequences through an open-response style, which more closely replicates the design of the original Corsi task than the multiple-choice style of the previous groups. They illuminated patterns on a screen with 10 blocks, which corresponded to 1 of 10 keys on a response pad. With 1 finger on each key, the subjects reproduced the sequence. This research team demonstrated distinct patterns of hippocampal connectivity in visuospatial processing by epilepsy type. They found that using the task to examine functional connectivity allowed for a novel and more direct assessment of the brain network abnormalities involved in visuospatial deficits in epilepsy (Doucet et al., 2013).

To our knowledge, no studies have examined the use of an fMRI-adapted Corsi task in PD patients or an older adult population. Consequently, in anticipation that the task may be similarly applicable to PD, this pilot study examines the feasibility of utilizing an adapted Block Span Task as an assessment of visuospatial deficits and mechanisms in individuals with PD across an older age range. Through a preliminary examination of neural activation patterns in a limited test sample of PD participants, this project aimed to determine whether the BST may be a practical and useful tool that allows for the integration of fMRI with a practical assessment of visuospatial cognition in adults with PD.

## 2 Methods

This study was approved by the Institutional Review Board of Emory University and the Review Committee for the Atlanta VA Medical Center. The trial is described in full in the clinicaltrials.gov registry item NCT04122690 and in the protocol report by Hackney et al. (2020). All participants gave informed consent prior to participation in this study.

### 2.1 Participants

Older adults with mild–moderate PD were recruited from the Atlanta area for a study examining the effects of exercise on PD symptoms. The data considered here concerns only baseline observations prior to beginning an exercise program. Participants were recruited through the Atlanta VAHCS Movement Disorders clinic, the VA Informatics and Computing Infrastructure (VINCI) database, the Michael J. Fox Foxfinder website, the Movement Disorders unit of Emory University, PD organizations’ newsletters, support groups and educational events, and word of mouth. Interested patients were provided with additional study information by telephone. One participant withdrew from the study prior to completing all assessments.

### 2.2 Inclusion and exclusion criteria

Participants were 49–82 years old, had a clinical diagnosis of PD (Hoehn and Yahr stages I–III), experienced “off” times with their anti-parkinsonian medication (score ≥1) on the Movement Disorders Society Unified Parkinson’s Disease Rating Scale (MDS-UPDRS) item 4.3 (i.e., time spent in off state) (Goetz et al., 2008) and were able to walk at least 10 feet with or without an assistive device. Exclusion criteria included diagnosis of dementia, vascular cognitive impairment, memory deficits, or other neurological disorders or insults, e.g., stroke. The Montreal Cognitive Assessment (MoCA) (Nasreddine et al., 2005) was used to screen out overt dementia. Participants were required to achieve a score of at least 18 to be enrolled (Hackney et al., 2020).

### 2.3 Assessments

As per Hackney et al. (2020) and for the study design of this inquiry, participants were assessed in one session for demographic and clinical characteristics and select measures of motor, cognitive, and psychosocial function with standardized, valid, and reliable assessments (Table 1). Participants were tested in the off state, at least 12 h following their last dose of antiparkinsonian medication, as has been considered good practice in studies examining the pathophysiology of PD (Gordon and Reilmann, 1999). Assessments included the MDS-UPDRS, the Freezing of Gait Questionnaire (FOGQ), the Physical Activity Scale for the Elderly (PASE), the Composite Physical Function Index (CPF), and the PD Questionaire-39 (PDQ-39) (Hackney et al., 2020). Participants self-reported comorbidities.

**Table 1:** Aggregate demographics of study participants.

Demographics/Clinical characteristics	Total sample *n* (%) or Mean ± SD
Age (years)	69.6 ± 8.1
Gender	
Male	12 (57.1%)
Female	9 (42.9%)
Race	
White/Caucasian	15 (71.4%)
Black/African American	5 (23.8%)
Multiracial	1 (0.05%)
Education	
High school/GED	2 (9.5%)
Some college/associate degree	6 (28.6%)
Bachelor’s degree	4 (19.0%)
Master’s degree	7 (33.3%)
Doctoral degree	2 (9.5%)
Total years	16.1 ± 2.4
Time with PD (years)	7.8 ± 5.7
Have fallen within the past 6 mo.	
Yes	8
No	12
Side of onset	
Left	11 (52.4%)
Right	10 (47.6%)
Number of comorbidities	3.4 ± 1.8
Hoehn and Yahr Stage (/5)	2.3 ± 0.5
MDS-UPDRS^a^	
Part I (nonmotor experiences of daily living, /16)	10.3 ± 6.5
Part II (motor experiences of daily living, /14)	12.6 ± 7.6
Part III (motor examination, /108)	38.6 ± 13.7
Part IV (medication-related motor fluctuations, /23)	6.2 ± 3.9
Freezing of gait (/24)	7.3 ± 5.9
Quality of life^b^ (/7)	5.5 ± 1.3
PASE total^b,c^	114.8 ± 80.7
CPF^b^ (/24)	20.3 ± 4.4
PDQ-39^a^	
Mobility score (/100)	16.4 ± 16.0
Activities of daily living (ADL) score (/100)	20.4 ± 19.1
Cognitive impairment score (/100)	24.7 ± 22.1
Handedness (writing)	
Right	18 (85.7%)
Left	2 (0.1%)
Unknown	1 (0.05%)
Average span achieved on other memory tasks	
Reverse Corsi (/9)	4.6 ± 1.3
BPST (/9)	3.9 ± 1.0
Number span- forward (/9)	7.3 ± 1.2
Number span- backward (/9)	5.0 ± 1.6
Average performance on other neuropsychological assessments	
Reverse Cori blocks product score (/112)	32.4 ± 13.2
Brooks spatial memory task % correct	73.6 ± 18.5
MOCA visuospatial/executive total (/5)	4.2 ± 0.8
MOCA total points (/30)	27.1 ± 2.5
Benson immediate recall score (/17)	15.3 ± 1.7
BPST total correct trials (/16)	4.3 ± 1.5
Benton JLO total correct trials (/15)	11.3 ± 3.4
Number span- forward total correct trials (/14)	9.2 ± 2.3
Number span- backward total correct trials (/14)	7.1 ± 2.7

*N* = 21.

^a^Higher scores indicate greater disability or severity of symptoms.

^b^Higher scores indicate better condition.

^c^Scores of sample of healthy older adults used in assessment development ranged from 0 to 360 (Washburn et al., 1993).

Participants also participated in tasks utilizing visuospatial abilities, including brooks spatial memory, reverse Corsi blocks, Benson complex Figure copy immediate, Benton’s judgement of line orientation (JLO), body position spatial task (BPST), and number span task (Hackney et al., 2020).

### 2.4 Block span task description and protocol

#### 2.4.1 BST implementation

**Figure 1:**
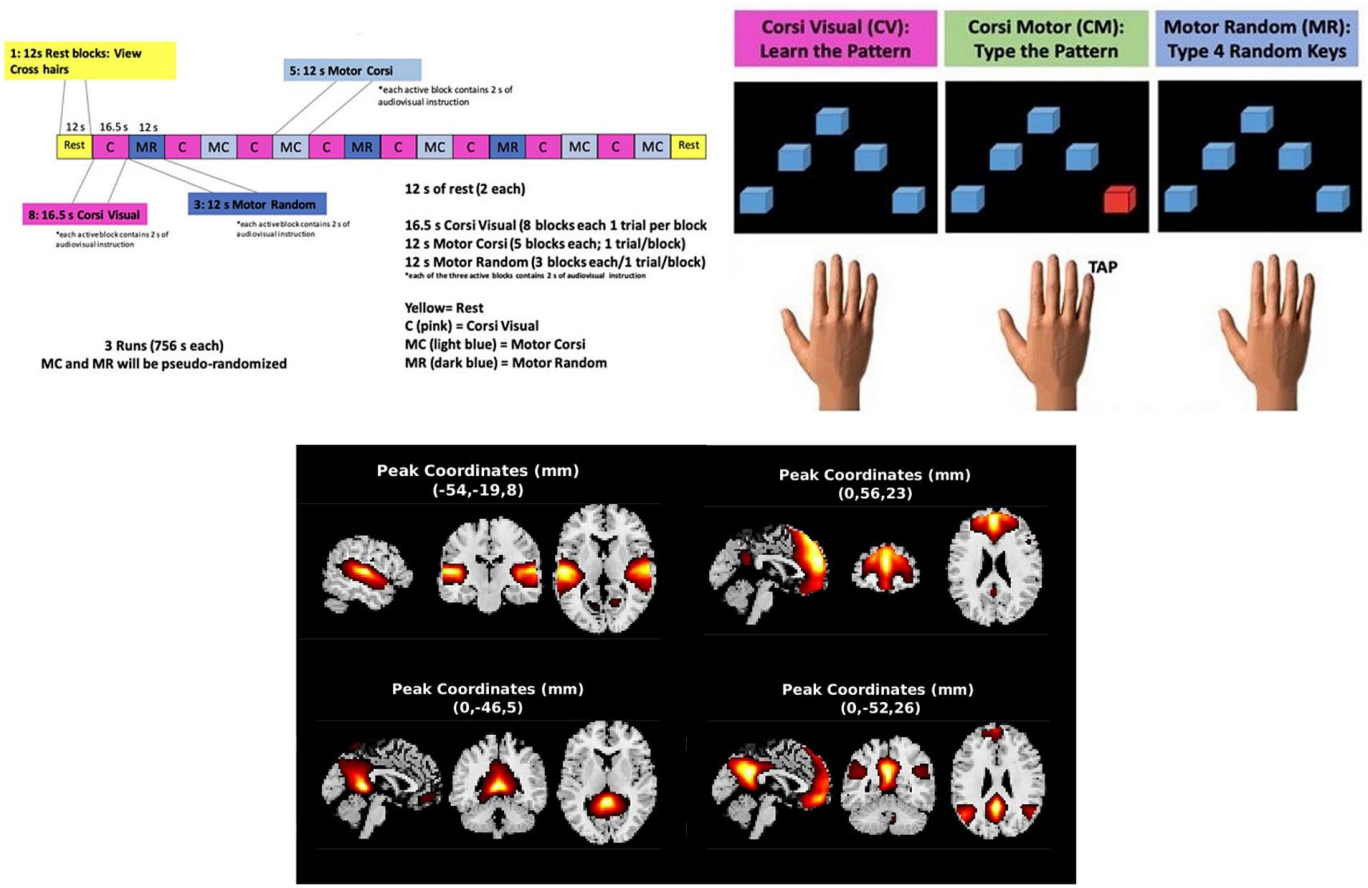
BST design and positively modulated components. Top left: Design of a single run of the BST; top right: BST interface; bottom: Components with positive significant beta values from Corsi visual-random motor contrast (clockwise from top left: Superior temporal gyrus, superior medial frontal gyrus, posterior cingulate cortex, precuneus).

Before the fMRI session, training ensured that each participant understood the instructions and could do the task. Within the MRI scanner, participants conducted the BST, programmed in ePrime3.0, wearing an MRI-compatible Celeritas response pad on their right hand. Participants participated in 3 task runs for adequate statistical power without tiring the participants. Each run included 8 Corsi Visual blocks, 5 Corsi Motor blocks, and 3 Random Motor blocks for a total of 24 visual sequence blocks and 24 motor sequence blocks (described below). A pre-baseline rest block of 12 s occurred before each run and a post-baseline rest occurred after the three runs (Figure 1). During the rest blocks, a hash sign was presented on the screen.

The in-scanner screen displayed 5 squares, and each square corresponded to 1 of 5 keys (one for each finger) on the Celeritas response pad (Figure 1). During Corsi Visual blocks (encoding periods), participants engaged in encoding processes as they were presented with audio and visual/text-based instructions that asked them to “Learn the pattern”, and individual squares on the screen were sequentially illuminated with a red color for 1.5 s each. The sequence patterns were pseudo-randomized and the sequence length for all participants was 4 squares. The reason for this length follows: A sequence length of 4 was determined by calculating the mean span (span = 4.2 ± 1.1) on the Corsi block task of 91 past participants with PD in prior work from the PI’s lab (Battisto et al., 2018). After each “Learn” period, the participants were presented with either a Corsi Motor period or a Motor Random period which were pseudo-randomized across runs. Before the Corsi Motor period, participants were presented with audio and text stating, “Type the pattern”. The participants engaged in retrieval as they replicated the block sequence learned during the Corsi Visual period with the response pad buttons. During the Motor Random period, participants were presented with audio and text stating, “Type random keys”. During this 12-s phase, participants chose to press 4 keys in the sequential order either from pointer finger to little finger or from little finger to pointer finger. During the Corsi Motor and Random Motor periods, the squares flashed red as visual confirmation when the participant pressed the corresponding key. Both motor phases lasted 12 s. Between sequences, an inter-stimulus interval (ISI) occurred with a jittered duration between 1.5 and 3 s. After the scan was completed, the participant was asked to describe any strategy that they used during the task to remember the pattern presented, and this information was documented (Table 2).

**Table 2:** Participant BST performance and motor measures.

Subject	BST percent of correct trials (%)	Handed-ness	Side of onset	R-hand: finger tapping	R-hand: hand movement	R-hand: postural tremor	R-hand: kinetic tremor	RUE: rest tremor amplitude	Strategy
1	93.3	R	L	1	1	1	1	0	Numbered the boxes
2	46.7	R	R	3	2	0	1	0	Numbered the boxes
3	80.0	R	L	1	2	0	1	0	Numbered the boxes
4	73.3	R	L	1	0	0	0	0	“Pictured the pattern to [their] fingers”
5	20 0.0	R	R	2	2	3	3	0	Tapped it out on her fingers
6	93.3	R	L	1	1	1	1	0	Numbered the fingers
7	60.0	L	L	2	2	2	2	0	“Spatial abstract thinking”
8	20 0.0	R	L	1.5	1	2	1.5	0	“Nothing in particular”
9	40.0	R	L	3	3	4	3	2	Numbered the boxes
10	53.3	R	R	3	3	1	1	3	“Watched screen and remembered where it was”
11	–	R	R	1	2	0	1	0	Numbered the boxes
12	46.7	R	R	1	1	0	1	0	–
13	20.0	R	L	2	2	1	2	2	“Remembered it as a number sequence”
14	–	R	R	1	0	1	0	1	–
15	6.7	R	R	1	1	0	0	0	–
16	46.7	L	L	1	2	0	1	0	“Tried to type/remember the pattern”
17	–	R	R	3	3	0	0	0	–
18	–	–	L	2	2	0	0	0	–
19	–	R	L	1	1	0	1	0	Numbered the fingers and keys
20	–	R	R	0	2	1	1	0	–
21	–	R	R	1	2	1	1	3	“Assigned numbers and generated a code to memorize”
Avg	50 ± 28	–	–	1.55 ± 0.86	1.67 ± 0.86	0.86 ± 1.11	1.07 ± 0.87	0.52 ± 1.03	
R-handed	49.4 ± 30.0								
L-handed	53.4 ± 9.4								
R-onset	34.7 ± 20.2								
L-onset	58.5 ± 28.6								

“R-hand: hand movement”, “R-hand: postural tremor”, “R-hand: kinetic tremor”, “RUE: rest tremor amplitude” scores taken from MDS-UPDRS items 3.4a, 3.5a, 3.15a, 3.16a, and 3.17a, respectively. Items scored on a scale from 0 (no problems) to 4 (severe problem). Score averaged if second rater present. Strategy data are presented as direct quotes where available; otherwise summarized by the study team.

#### 2.4.2 Image acquisition

Neuroimaging data were collected on a 3 T Siemens Prisma Fit scanner using a 32 Channel Head coil. Participants completed three functional scans of the BST, using a multi-band T2* echo planar imaging sequence with the following acquisition parameters: (TR) = 1,500 ms, echo time (TE) = 25.00 ms, flip angle = 50 degrees, bandwidth = 2,164 Hz/pixel, field of view (FOV) = 220 mm, matrix = 110 × 110, voxel size = 2.0 × 2.0 × 2.0 mm, 72 continuous slices, with interleaved acquisition, multi-band = 3, iPAT acceleration factor = 3, 168 volumes, acquisition time: 4:35 min per run, phase encoding direction = AP. An anatomical image was collected using a high resolution MPRAGE scan sequence (TR = 2,400.0 ms, TE = 2.72 ms, flip angle = 50 degrees, FOV = 256 mm, matrix = 300 × 320, bandwidth = 210 Hz/pixel, voxel size = 0.8 × 0.8 × 0.8 mm, with 208 contiguous slices in the sagittal plane).

#### 2.4.3 Neuroimaging data pre-processing

Task-based fMRI data pre-processing was performed in BrainForge, a cloud-enabled, web-based analysis platform for neuroimaging research (Verner et al., 2023). For each participant, all fMRI data was first converted from DICOM to NIfTI using dcm2niix (v1.0.20211006) (Li et al., 2016). Each of three fMRI runs were analyzed with an in-house containerized version of SPM 12 (v7018), using a standardized analysis pipeline. Images were first corrected for EPI distortion using a reverse phase encode fieldmap. FSL’s (v 6.0.3) topup was run to estimate field distortions and applytopup was used to apply the corrections to the data (Andersson et al., 2003; Smith et al., 2004). Slice time correction was applied to correct time-shifts due to multi-band acquisition. Data were motion-corrected as necessary in one of three translational directions (*x*, *y*, *z*) and/or three rotational axes (pitch, roll, yaw) before warping to a standardized TPM (probability map) template in MNI space.^1^ Warped images were smoothed using a 6 mm FWHM Gaussian kernel (Ashburner et al., 2021). As an added quality assurance step, plots of each of the estimated motion parameters were assessed visually. As well, mean framewise displacement (FD) indices were calculated for each run. Mean FD values greater than 0.5 mm were considered indicative of severe head motion (Power et al., 2012). None of the subjects were excluded based on this criterion.

### 2.5 Analysis

Descriptive statistics and means were computed for the demographic and clinical characteristics and performance on neuropsychological assessments by all participants (Table 1). For each participant, the number of correct response sequences and the percentage of trials with correct responses were calculated across the three runs (Table 2).

#### 2.5.1 Neuroimaging data

The pre-processed data were entered into the GIFT software package (Group ICA of fMRI data Toolbox, version 4.0b)^2^ to perform spatially constrained independent component analysis (ICA) with 53 pre-defined component maps using the NeuroMark_fMRI_1.0 template^3^ (Du and Fan, 2013; Du et al., 2020). NeuroMark template maps are used as spatial priors to compute independent component spatial maps and timecourses for each participant/acquisition session. This approach is fully automated and allows to preserve correspondence of components between participants, while also optimizing for individual participant maps and timecourses.

We used the MOO-ICAR ICA algorithm with GICA2 back-reconstruction, removing mean per time point, applying a high-frequency filter with cut off frequency of 0.15 Hz (Erhardt et al., 2011; Meng et al., 2023). The timecourses were despiked using the GIFT despiking procedure, which is conceptually similar to AFNI’s 3dDespike method (MAD-based detection of outliers relative to a smooth baseline fit, with nonlinear attenuation of extreme values) (Cox, 1996; Cox and Hyde, 1997). The independent components (ICs) were temporally sorted using multiple regression with a design matrix based on the 3 regressors used during task fMRI data acquisition: Corsi Visual (reflecting the encoding phase), Corsi Motor (reflecting the retrieving phase), and Random Motor (reflecting the control condition). Stimulus onset timings and participant response timings were closely aligned in time across all participants and sessions; therefore, the same timing was used across all datasets. Thus, using the task-based design matrix, we performed multiple linear regression to correlate IC timecourses with the model timecourses constructed using the three regressors above. This process allowed for computation and statistical analysis of the beta weights, also known as the slopes of the regressors, for each of the tasks. Beta weights reflect the degree to which the fMRI activity for a given component was modulated by the task condition.

False Discovery rate (FDR)- adjusted *p*-values were calculated to assess the significance of the difference in beta weights for each of the contrasts. For significance criterion, *a* = 0.05 was selected. This study examined the components with positive significant beta values, which indicated that the activity of that component was more associated with the Corsi Visual or Corsi Motor phase, the condition of interest, than the Random Motor phase, the control-like condition.

#### 2.5.2 Imaging analysis: Corsi motor minus random motor

This analysis was conducted to reveal the brain networks modulated during the retrieving phase of the WM, controlling for the motor component. In detail, the difference in beta weights between the Corsi Motor regressor and Random Motor regressor (averaged across the 3 runs) was computed, and entered into a one-tail one-sample t-test to determine whether and which brain networks were positively modulated by the Corsi Motor phase, compared to the control condition (Random Motor).

#### 2.5.3 Imaging analysis: Corsi visual minus random motor

This analysis was conducted to reveal the brain networks modulated during the encoding phase of the WM, controlling for the motor component. As was performed for the retrieving phase’s Corsi Motor and Random Motor regressors, the difference in beta weights between the Corsi Visual and Random Motor regressor (averaged across 3 runs) was computed, and entered into a one-tail one sample t-test to determine whether and which brain networks were positively modulated by the encoding phase (Corsi Visual), compared to the control condition (Random Motor).

For both encoding and retrieving phase contrasts, the Random Motor phase was treated as a control to account for nonspecific motor functions during Corsi Motor and for any extraneous or involuntary motor activity during Corsi Visual including tremor, a possibility indicated by the nonzero tremor scores of several participants. The Random Motor condition should also represent other baseline mental processes such as basic visual function when viewing the screen, which, when subtracted from the Corsi Visual or Corsi Motor condition, contributing to a more unobscured representation of encoding-specific processes.

## 3 Results

### 3.1 Participant clinical characteristics and neuropsychological assessments

Participants’ disease severity was mild to moderate in MDS-UPDRS nonmotor and motor assessments (Goetz et al., 2008), and the average PASE score indicated ordinary physical activity levels (Washburn et al., 1993). The average CPF score indicated participants were at moderate risk for lost function (Rikli and Jones, 2012) (Table 1). Participants scored low on freezing of gait measures and self-rated relatively high on quality of life. The average PDQ indicates that participants had greater burden to health-related quality of life in cognitive impairment than ADLs, and in ADLs than mobility. Individual handedness, side of PD onset, right hand tremor assessments, and right hand and finger usage assessments are shown in Table 2.

Participants achieved an average Reverse Corsi product score within 1 standard deviation of the average performance by a sample of healthy adults (Kessels et al., 2008). Their total correct BPST trials, representing whole body spatial cognition, was also not below that of healthy adults (Battisto et al., 2018). Their percent accuracy was relatively high on the Brooks Spatial Memory Task. The participants’ average MoCA score was above the cutoff for MCI (Nasreddine et al., 2005). The participants’ performance on the Benson Complex Figure- Immediate assessment and the Benton JLO were also within 1 standard deviation of that found in healthy populations, and participants achieved a total number of correct trials on the Number Span tests consistent with that of healthy adults (Litvan et al., 2012).

### 3.2 Block span task performance

Percents of correct BST trials and strategy for each subject are listed in Table 2. BST performance data could not be collected for 7/21 participants due to technical errors in data collection with the Celeritas response pad.

### 3.3 Imaging analysis: Corsi motor minus random motor

After correcting for multiple comparisons using a FDR correction, no components showed significant modulation by the Corsi Motor vs. Random Motor contrast.

### 3.4 Imaging analysis: Corsi visual minus random motor

After FDR correction, 4 components showed a positive significant modulation by the Corsi Visual, compared to the Random Motor condition (Table 3, Figure 1). These included networks across three domains: auditory (superior temporal gyrus), cognitive control (superior medial frontal gyrus) and default-mode (precuneus, and posterior cingulate cortex).

**Table 3:** Significant components of Corsi visual minus random motor contrast.

Component ID	Component name	Peak coordinates (mm)	Average beta value	FDR adjusted P-value
Auditory domain				
6	Superior temporal G	(−54, −19, 8)	1.01	0.0131
Cognitive control domain				
28	Superior medial frontal G	(0, 56, 23)	1.59	0.0128
Default mode domain				
44	Precuneus	(0, −46, 5)	1.22	0.0177
49	Posterior cingulate cortex	(0, −52, 26)	2.06	0.0075

Table shows components with positive beta weights that were significantly modulated by the Corsi Visual condition after removing the Random Motor Condition. Average beta values were calculated from Corsi Visual—Random Motor.

## 4 Discussion

This pilot study investigated an adapted Block Span Task using fMRI in older individuals with PD. While the sample size is relatively small, the results suggest that it could be a useful tool for the investigation of visuospatial WM in this population, for the task appeared to show recruitment of various relevant brain networks supporting the encoding phase of the task. In detail, during the encoding phase of the task, the task appeared to show a positive modulation of four components covering the superior temporal gyrus, superior medial frontal gyrus, precuneus, and posterior cingulate cortex. During the memory retrieval phase of the task, no components showed significant modulation.

### 4.1 BST strategy

The strategies reported by participants warrant consideration. The participants who utilized a numbering strategy would have participated in a WM task that is perhaps more reminiscent of the Number Span Task than the Corsi Blocks. The usage of this strategy may have consequently reduced the degree of visuospatial involvement in this task for those participants, as rather than only remembering a visual sequence of blocks in space, they could instead memorize a non-visual sequence of numbers. However, using a numbering strategy does not preclude the involvement of visuospatial processes as participants must still map the numbers to each block’s unique spatial context and to their fingers, which participants must line up to the blocks by their spatial arrangement.

### 4.2 Imaging analysis: Corsi visual–random motor

For this contrast, our main results revealed networks part of major cognitive domains such as the default mode and cognitive control and sensorimotor domains. With regard to the default mode domain (DMN), we saw two posterior components positively modulated by the Corsi Visual phase, encompassing the posterior cingulate cortex (PCC) and precuneus. The DMN supports internally-directed processing and its activity is known to decrease during externally-directed or goal-directed tasks. Thus, the positive modulation of the PCC and precuneus during the goal-directed encoding of the BST was unexpected. A potential explanation lies in the altered DMN function and connectivity found in PD patients; PD patients have shown less DMN deactivation during externally-directed tasks (Wang et al., 2025). Alternatively, the DMN has also shown increased connectivity as a compensatory mechanism in cognitive impairment (Gardini et al., 2015). Beyond patient populations, PCC activity has been linked with increased states of arousal or awareness and to correlate with efficiency of cognitive processing in some contexts (Leech and Sharp, 2014). In some externally-directed tasks, increased PCC activity correlates with improved performance such as faster reaction times to unpredictable stimuli (Leech and Sharp, 2014), a context which could relate to the BST as the participants cannot predict the sequence they will be presented with during the encoding period. Other studies have demonstrated the PCC’s role in visuospatial cognition. One study showed increased PCC activation during eye movement tasks (Kravitz et al., 2011), which could be relevant to this contrast if participants engaged in more eye movement or scanning of the stimuli during the Corsi Visual period to not miss any part of the sequence when learning the sequence. That study also showed that PCC activity increases with demand on spatial attention selection (Kravitz et al., 2011), which is similarly relevant to this study’s encoding phase as participants must shift their attention between the different spatial locations as each of the blocks is illuminated in sequence. The precuneus modulation in this contrast can also be explained by its known activation associated with spatial location encoding, holding spatial information in WM (Frings et al., 2006). The precuneus is also involved in shifting visuospatial attention (Cavanna and Trimble, 2006), which, like the PCC, is required in the BST as participants track the different blocks as they are illuminated. Alternatively, this positive modulation could reflect either compensatory recruitment or just poor performance in the patients. Larger studies are warranted to determine the origins of this finding. Altogether, the unexpected modulation of the PCC would have notable implications if future study should confirm this as an area of difference in PD patients through comparison to the administration of the BST in a control group.

We also revealed a network encompassing the superior medial frontal gyrus from the cognitive control domain responding positively to the encoding phase. The superior medial frontal gyrus includes the supplementary motor area (SMA) and presupplementary motor area. The SMA may help inhibit motor activation when a motor plan can be created but no physical motor action is required (Nachev et al., 2008). This role may be relevant for the encoding phase because as they watch the blocks illuminate, participants may form motor plan for their corresponding finger, but because they are not meant to press the buttons during this phase, the SMA could be recruited to inhibit activation of those motor processes. Another possible avenue for the superior medial frontal involvement is its demonstrated activation in conditions where a subject participating in a motor task is presented with a change in action selection rules or when a subject is presented with a cue that indicates an impending motor task (Rushworth et al., 2004). Significant modulation of the superior medial frontal gyrus in this contrast can be attributed to the fact that these conditions could be used to describe the encoding phase of the BST as participants are learning a new sequence that will dictate their impending motor responses. Lastly, studies have shown increased neurophysiological response of the superior temporal gyrus with greater phonological WM load (Perrachione et al., 2017). As the participants who reported a numbering strategy would have been utilizing phonological WM, the temporary storage of verbal information, the significant modulation of the superior temporal gyrus in this contrast is relatively consistent with the expectations for this phase.

### 4.3 Imaging analysis: Corsi motor–random motor

For our analysis involving the retrieval phase, no significant components were revealed after a multiple-comparison correction was applied, contrary to expectations. Prefrontal and parietal cortices and BG are known to be involved in WM, and the brain regions involved in sensory processing have also been shown to contribute to the storage of WM of that modality (Eriksson et al., 2015). The unexpected results of this analysis could be attributed to limitations of the study.

### 4.4 Limitations

Limitations exist for this study. The average accuracy on the BST (50% in average) was relatively low, which could suggest that the BST was too difficult for the patients, and led to a lack of activation. However, average spans near or above 4 on the memory tasks including the original Corsi indicate that this population was able to hold at least 4 visuospatial items in their WM. As participants’ average overall scores on other WM tasks were on par with healthy populations suggests that the BST, a task of similar difficulty and span, would not exceed these participants’ abilities to accomplish. Furthermore, participants with the lowest scores on the BST did not consistently fall more than one standard deviation below the average span or score on the neuropsychological assessments or memory tasks. The participants also exhibited, on average, generally slight or mild tremor symptoms and right upper extremity motor difficulties, but it is unclear whether this degree of symptom could affect their ability to correctly operate the response pad. Some of the lower-scoring participants on the BST did have greater levels of tremor and motor difficulties in their right arm, hand, and/or fingers, than the average, so these symptoms may be responsible for their poor performance on the BST compared to the other tasks which did not require relatively fine motor skills of the right hand. Future implementation of the BST may reduce the influence of these concerns by offering participants the option to use a left-handed response pad if they experience more effective motor operation of that hand.

Altogether, the novelty and stress of being in an fMRI scanner may have impacted subjects’ performance on the BST compared to the other tasks which took place in standard office conditions. Future studies should consider quantitatively investigating any potential relationship between accuracy on the BST and accuracy on WM tasks outside of the scanner. In general, low performance was also a concern shared by Doucet et al. (2013) in their own BST, which limits the conclusions that can be drawn from this data regarding visuospatial WM, for the imaging results corresponds to encoding or retrieval processes that were not highly accurate.

With regards to the unexpected, apparent lack of modulation by the Corsi Motor vs. Random Motor contrast, the Random Motor condition may not have fully isolated motor from cognitive processes, as patients may have used overlapping and identical resources for Corsi Motor and Random Motor conditions; in the Random Motor condition, participants had to remember to enter the index-to-pinky or pinky-to-index sequence, which could have been cognitively tasking, potentially reducing its difference in mental processes compared to the Corsi Motor phase. Future adaptations of the BST should consider altering the Random Motor instructions to reflect a more truly random condition such as “Press any four keys.”

The use of a numbering strategy and potential engagement of phonological WM by some participants, including those with the highest accuracy on the BST, reveals another weakness of this study’s BST as the simplicity of its simple 5-block arrangement lends more easily to number assignment than the scattered, 9-block arrangement of the original Corsi task or the Doucet study’s 10-block arrangement (Doucet et al., 2013) in which labeling the blocks would have a higher ceiling effect. Future use of the tool may consider altering this aspect of the design to better isolate WM.

Finally, given this study’s primary focus on implementation of the task, conclusions regarding the imaging findings are limited because of the relatively small sample size and the lack of a healthy control group. In particular, because the lack of healthy participants limits our ability to interpret findings as PD-specific, future study should include a control population in addition to a larger PD sample size. Similarly, future study should add age as a covariate in statistical models.

## 5 Conclusion

Impairment in visuo-spatial WM remains a serious factor in living with PD. Existing literature has consistently indicated that cognitive and neuropsychiatric deficits in PD affect social functioning and activities of daily living (Chen et al., 2022) and such cognitive-driven functional decline predicts progression to dementia (Becker et al., 2022). The lack of treatments that are effective in mitigating non-motor symptoms compared to motor symptoms indicates continued need for more understanding of the underlying mechanisms. The integration of the Corsi blocks task and fMRI allows the BST to take advantage of the expansive knowledge base behind a popular, validated task as context for measurements obtained with the additional potential offered by imaging technology. Other attempts at Corsi-fMRI implementation have utilized an approach with a less faithful replication of the Corsi task or have not specifically examined its use in an older PD population. This study was designed to be a pilot test examining the usage, practicality, and potential value of our BST design to investigate WM in this particular patient population. The imaging results suggest that the BST can be used to engage certain WM networks such as the superior medial frontal gyrus as expected and designed. It also hints at potential areas of difference in the PCC in PD patients, which the BST can be used to further investigate in a future study. Thus, although the BST warrants further refinement, it exhibits appreciable potential for future use as an assessment tool of the neural mechanisms underlying visuospatial skills in this population of older individuals with mild–moderate PD.

## Data Availability

The raw data supporting the conclusions of this article will be made available by the authors, without undue reservation.

## Footnotes

1
https://www.fil.ion.ucl.ac.uk/spm/toolbox/TPM

2
https://github.com/trendscenter/gift%20with%20Matlab%202016b%20Runtime

3
https://trendscenter.org/data/
